# Effects of exenatide on coronary stent’s endothelialization in subjects with type 2 diabetes: a randomized controlled trial. The Rebuild study

**DOI:** 10.1186/s12933-023-02071-4

**Published:** 2023-12-08

**Authors:** Irene Santos-Pardo, Nils Witt, Oskar Angerås, Thomas Nyström

**Affiliations:** 1 Department of Clinical Science and Education, Karolinska Institute, Unit of Cardiology, Södersjukhuset, Stockholm, Sweden; 2 Department of Molecular and Clinical Medicine, Institute of Medicine, University of Gothenburg, Gothenburg, Sweden; 3 Department of Cardiology, Sahlgrenska University Hospital, Gothenburg, Sweden; 4 Department of Clinical Science and Education, Unit of Internal Medicine, Karolinska Institute, Södersjukhuset, Stockholm, Sweden; 5 Department of Cardiology, Södersjukhuset. Sjukhusbacken 10, 11883 Stockholm, Sweden

**Keywords:** Type 2 diabetes, Glucagon-like peptide-1 receptor agonists, Exenatide, Stent endothelialization, Drug eluting stent

## Abstract

**Background:**

Subjects with type 2 diabetes (T2D) have a higher risk of in-stent restenosis and stent thrombosis. The activation of the glucagon-like peptide-1 receptor (GLP-1R) has been suggested to induce several effects on the vasculature that may reduce the risk of stent failure following an angioplasty. The aim of this study is to evaluate the effect of the GLP-1R agonist exenatide on endothelialization of a modern drug-eluting stent (DES) in subjects with T2D.

**Methods:**

38 subjects with T2D who were eligible for revascularization with implantation of DES were randomized to treatment with exenatide (once weekly) plus standard treatment, or to standard treatment alone. After 12 weeks, a new coronary angiography was performed to evaluate the percentage of strut coverage (primary endpoint) and the presence of neo-atherosclerosis by optical coherence tomography. This study was approved by the Stockholm’s Ethical Review Board.

**Results:**

The two groups were well balanced regarding baseline clinical characteristics. Strut coverage was 95% (88.7–98.5%) in the exenatide group and 91.4% (88.8–98.5%) in the control group (p = 0.692). There were no significant differences between groups neither in the thickness of neo-intima (0.2 mm in both groups, p = 0.471), nor the maximal in-stent obstruction by neo-intima (15.5% in exenatide group *vs* 14.7% in control group, p = 0.801). No significant differences were detected in the rate of target lesion revascularization between groups (p = 0.224).

**Conclusion:**

Twelve weeks treatment with exenatide did not lead to a significantly better stent coverage in people with T2D. No significant differences in the occurrence of neo-atherosclerosis were detected between groups.

*Trial registration:* The study was registered at www.clinicaltrials.gov (Rebuild Study, NCT02621489).

**Supplementary Information:**

The online version contains supplementary material available at 10.1186/s12933-023-02071-4.

## Introduction

Cardiovascular (CV) disease remains the leading cause of mortality among people with diabetes, accounting for forty percent from coronary heart disease (CHD) [1]. Coronary revascularization is, together with optimal anti-ischemic therapy, the cornerstone of ischemic heart disease (IHD) treatment. Although the introduction of second generation drug-eluting stents (DES) have offered the combination of reduced rates of in-stent restenosis (ISR) and lower risk for stent thrombosis (ST), [2] subjects with diabetes still have a worse prognosis following revascularization with percutaneous coronary intervention (PCI) compared to people without diabetes [3].

Deployment of a stent causes vascular wall injury characterized by endothelium denudation, disturbing the balance of numerous aspects of endothelial function that may ultimately lead to thrombosis and neo-atherosclerosis [4]. Studies demonstrate that activation of the glucagon like peptide-1 receptor (GLP-1R) induce proliferation of coronary endothelial cells, [5] inhibits the migration of circulating monocytes into the artery wall [6] and decreases the proliferation [7] of vascular smooth-muscle cells; effects that may represent a mechanistic model for reduction of ISR and ST.

Intracoronary Optical Coherence Tomography (OCT) is an intra-coronary imaging technique with an exceptional axial resolution of 10–15 µm which provides a near-histology level for detection and quantification of neo-intimal growth over the DES’ struts [8]. The aim of this study is to evaluate whether GLP-1R agonist exenatide improves DES endothelialization (percentage of strut coverage) in people with T2D undergoing coronary stent implantation.

## Methods

### Study design

Randomized, comparator-controlled, open label, assessor-blinded multicentric trial (Södersjukhuset in Stockholm, Sweden and Sahlgrenska University Hospital in Gothenburg, Sweden). The European Cardiovascular Research Center (CERC) core lab analyzed the OCT-derived endpoints and was blinded to the patient’s identity, allocation arm and the baseline clinical characteristics. The CERC is a core lab totally independent from our research institution. The study was approved by the Ethical Review Board of the Stockholm County Regional Council, the Medical Products Agency and the Swedish Radiation Safety Authority and complies with the current ICH E6 (R2), good clinical practice guidelines and the Helsinki declaration.

### Study population

**Fig. 1:**
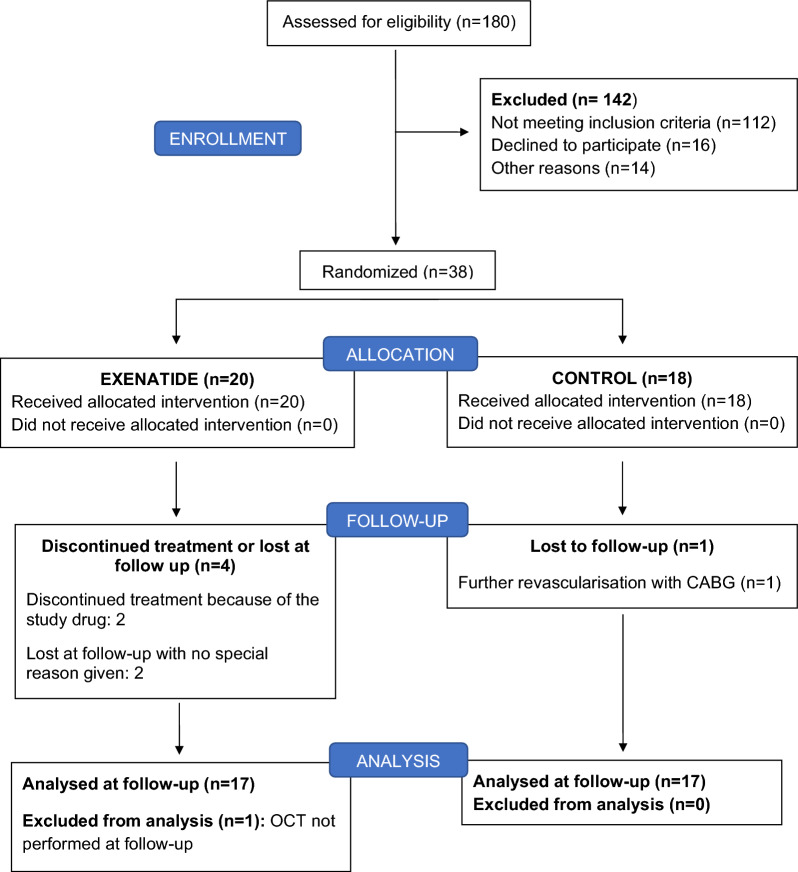
Study participants flow chart. Participants enrolled and finally analyzed are shown in the figure. *CABG* Coronary Artery By-pass Grafting, *OCT* Optical Coherence Tomography

We aimed to include female and male subjects aged 18 to 80 years old with known or newly diagnosed T2D with a HbA1c between 47–110 mmol/mol (DCCT 6.5–12.2%), who were eligible for PCI with implantation of DES. Indication for PCI included stable angina, unstable angina and non-ST elevation myocardial infarction (NSTEMI). Exclusion criteria are listed in Additional file 1: Table S1. Figure 1 shows the study inclusion flow. Screening failure summary is available in Additional file 1: Table S2.

### Study procedures and exposure

**Fig. 2:**
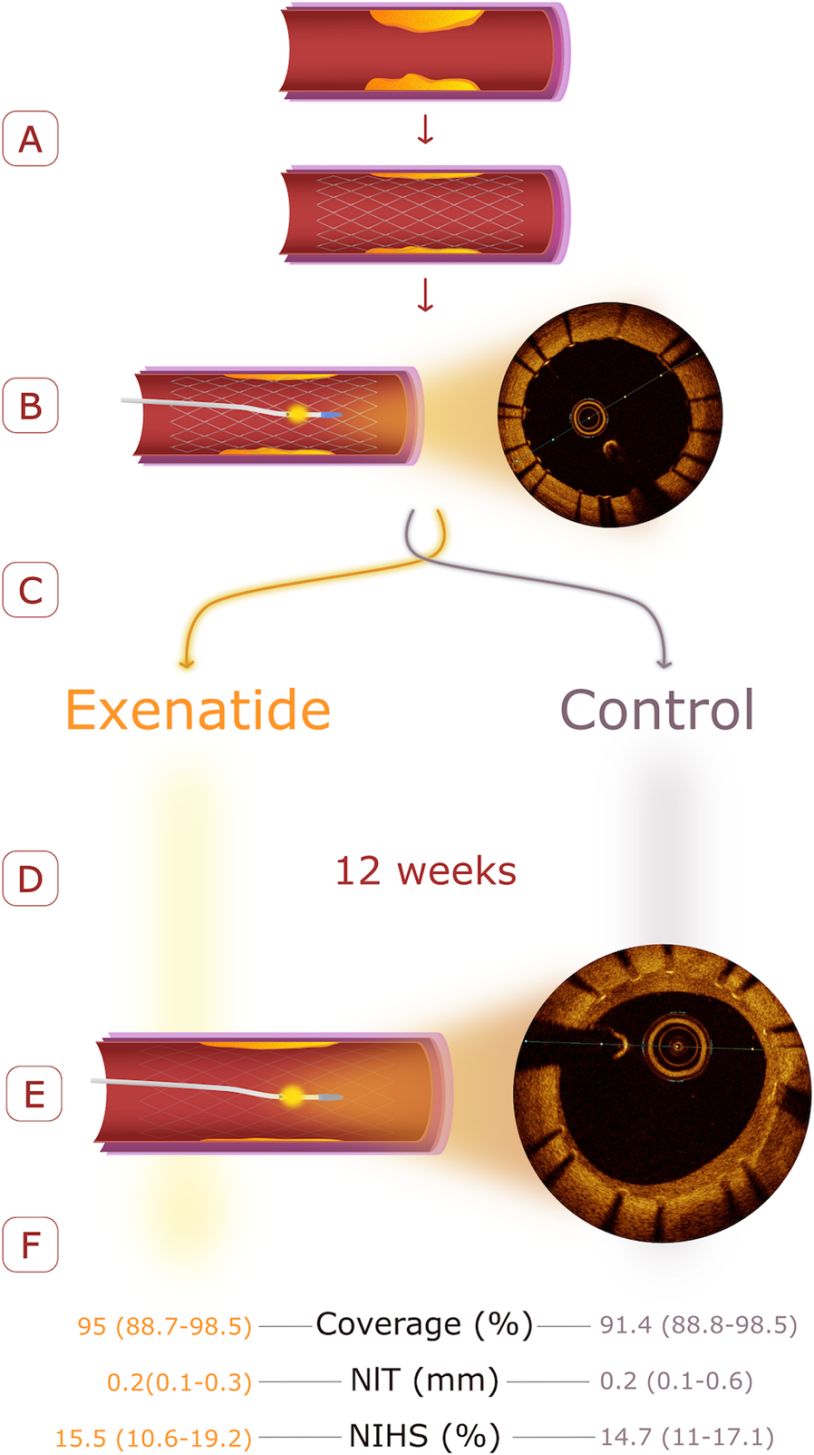
Methodology and main results. Panel A to C summarizes the methodology of the study. **A**: A coronary stenosis in a patient with T2D suitable for stenting is found, dilated, and stented with a Medtronic’s Resolute Onyx^®^ DES. Then, OCT is performed to ensure optimal stent expansion and vessel wall apposition. **B**: Participants are randomized to either exenatide (subcutaneous injection once weekly) over standard treatment or standard treatment alone. During the 12 weeks of treatment patients are contacted twice and are interviewed to detect possible adverse events. **C**: A new coronary angiography including an OCT of the region of interest is performed to assess strut coverage, luminal and stent measurements as well as neo-intima measurements. **D**: Main results for each treatment group. *DES* Drug eluting stent, *OCT* optical coherence tomography, *T2D* type 2 diabetes, *NIT* neo-intimal thickness, *NIHS* neo-intima hyperplasia stenosis

PCI was performed including a post-PCI OCT recording. Thereafter, participants were randomized either to the study drug exenatide and standard treatment, or standard treatment alone and were followed for 12 weeks. During these 3 months, 2 telephone visits were held to ensure participant safety. Study drug adherence was controlled by counting pens and tablets. At 12 weeks, a new coronary angiography including OCT examination was performed to assess strut coverage and neo-intimal growth. Figure 2 provides a summary of the methods of the study.

#### Catheterization protocol, intracoronary measurements, and angioplasty

Cardiac catheterization was performed following local standard procedures; radial access was preferred if no contraindications existed, all patients were on chronic treatment with acetylsalicylic acid or were given a loading dose of 300 mg the day before the procedure and all patients were loaded with a P2Y_12_ inhibitor (ticagrelor or clopidogrel) before PCI. Routine angiography cines were acquired for complete anatomic evaluation and stenosis severity was visually assessed. If considered necessary, intracoronary physiologic assessment with fractional flow reserve was performed to establish functional lesion severity. At the decision of PCI, 0.75 mg/kg enoxaparine or 50–100 E/kg unfractioned heparin was administrated. Six French guide catheters were used for angioplasty and OCT recordings.

##### Stent implantation

Implantation technique was chosen at the operators’ discretion, but maximal stent expansion including post-dilatation with non-compliant balloons was encouraged in all cases. All patients received a Resolute Onyx^®^ DES (Medtronic, Minneapolis, MN, US), which has a single wire platform with 81 µm rounded strut cross-sections, a platinum-iridium core, and a cobalt-chrome shell. Resolute Onyx^®^ DES elutes zotarolimus through a durable polymer (BioLinx) [9]. This stent was chosen for its known good performance in terms of arterial healing, having low rates of malapposition and high percentage of strut coverage in previous observational studies [10].

##### OCT image acquisition protocol and analysis

After stent implantation, intracoronary frequency domain-OCT recordings were obtained using the commercially available ILUMIEN OPTIS™ or OPTIS integrated™ systems with the Dragonfly™ rapid exchange OCT catheter (Abbott, St Paul, MN, US). Intracoronary nitroglycerine was administered before starting the pullback. Blood displacement was achieved by manual injection of contrast and pullback speed was set to 54 mm/s. If suboptimal stent result such as malapposition were found in the immediate post-PCI OCT, further optimization was performed, and a new OCT was recorded. At follow-up, OCT recordings were obtained following the same protocol. OCT recordings were analyzed with Caas IV-LINQ software version 2.1 (Pie Medical Imaging Systems, Maastrich, The Netherlands) with a frame slice thickness of 0.1 mm for 54 mm/s pullbacks and 0.2 mm on the occasions where pullback speed was set to 75 mm/s.

#### Exposure

After PCI, participants were randomized to either exenatide, i.e. Bydureon^®^ 2 mg once weekly in subcutaneous injection plus standard treatment or standard treatment alone. Standard treatment was defined as metformin (target dose 1 g bid) and Neutral Protamin Hagedorn (NPH) insulin (subcutaneous injection at bedtime and dose-adjusted for every specific case to achieve fasting glucose levels of 6 mmol/L). This was to ensure an optimal and comparable glycemic control throughout the study population. Drug-naïve patients were given NPH insulin and metformin at randomization and were up-titrated to achieve target glycemic control or the maximal tolerated dose, i.e. metformin. Participants who were already insulin-treated continued with their existing insulin given that a good glycemic control had been previously achieved.

#### Follow-up

During the 12 weeks of follow-up, participants were asked to keep record of capillary glucose measurements. Four and eight weeks after randomization, telephone visits were held to review the self-reported glucose measurements. At 12 weeks, repeat coronary angiography with OCT examination was performed as detailed above.

### Outcomes

#### Primary outcome

**Fig. 3:**
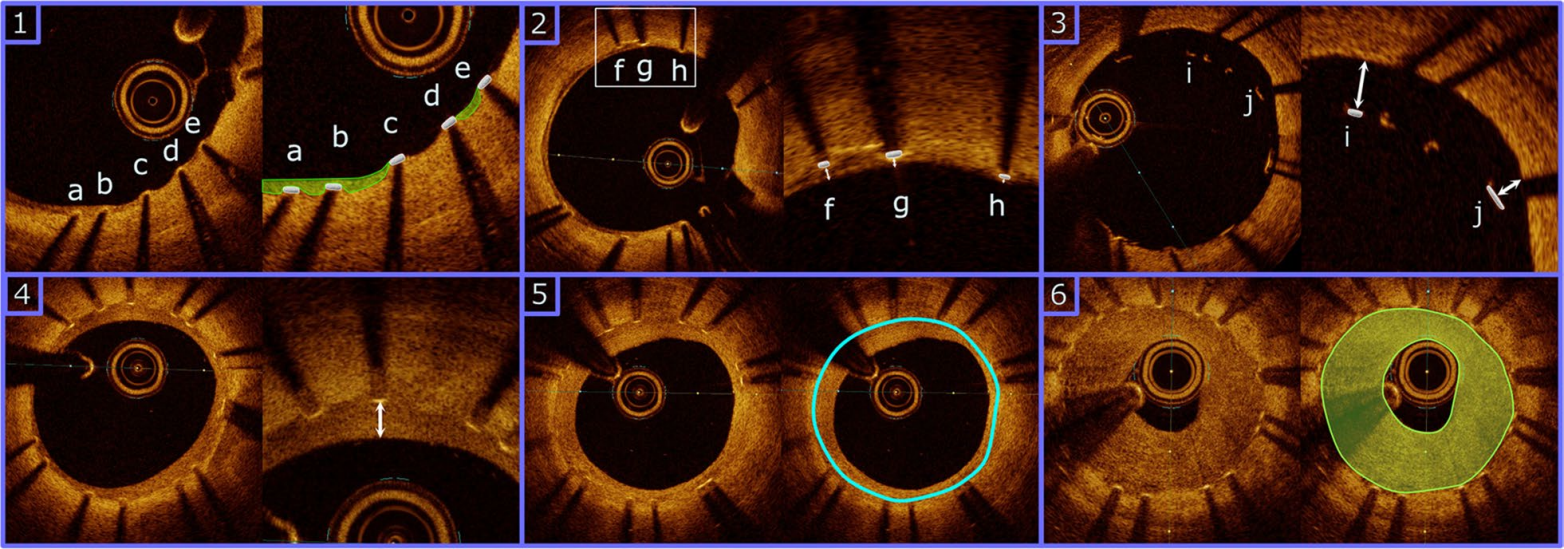
Definitions of OCT derived endpoints. In the schematic images struts are represented as boxes with metallic gradient filling. NI is represented as delimited translucid green areas. Panel 1: Stent strut coverage. A well-apposed stent covered by a thin NI layer. Struts a and b are considered covered by NI while struts c, d and e are classified as un-covered. Panel 2: Coverage thickness. Detail of 3 struts with different NI coverage thickness. NI coverage is measured as 100 µm for strut f, 60 µm for strut g and only 30 µm for strut h. Strut h would be considered covered for the primary endpoint but uncovered for the > 40 µm coverage thickness (secondary endpoint). Panel 3: Significant malapposition. An area of stent malapposition is seen from 11 to 2 o’clock. The abluminal side of strut i is separated 440 µm from the vessel wall and classified as significantly malapposed. Strut j is separated 260 µm from vessel wall which classifies for non-significantly malapposed. Panel 4: NI thickness. NI thickness measures 270 µm from the luminal side of the strut to the luminal border. Panel 5: Stent area. Seeable struts delimit stent area displayed as a light blue line. Panel 6: NI hyperplasia stenosis is calculated as NI volume (NI area multiplied by frame thickness) divided by stent volume (stent area multiplied by frame thickness) multiplied by 100. *OCT* Optical coherence tomography, *NI* Neo-intima

The percentage of stent strut coverage as assessed by OCT. A strut was deemed covered if tissue was identified above the struts (Fig. 3, Panel 1).

#### Secondary outcomes

##### OCT derived endpoints

The following endpoints were analyzed: percentage of strut coverage > 40 µm, stent strut significant malapposition (distance between the luminal part of the strut to intimal border > 300 µm), malapposition volume, mean neo-intimal area, neo-intimal hyperplasia area at maximal obstruction, percentage of stenosis by neo-intimal hyperplasia, neo-intimal hyperplasia volume at maximal obstruction and maximal neo-intimal thickness at maximal obstruction. Luminal and stent areas and volumes as well as stent expansion were measured at baseline and follow-up. Definitions for some selected OCT derived endpoints are given in Fig. 3. Remaining definitions for OCT endpoints are found in Additional file 1: Table S3.

At baseline, visual assessment of plaque composition, calcium characterization and the presence of thrombi and tissue protrusion was performed for the site of maximal obstruction (culprit lesion) as well as proximal and distal vessel references (within 5 mm to stent edges). The total number of struts analyzed per lesion and the mean number of struts per cross-section were also reported.

##### Clinical endpoints

Need for target lesion revascularization (within 12 weeks or at follow-up angiography). Anthropometric and biochemical endpoints were pre-defined as changes between baseline and follow-up: body weight, abdominal circumference, blood pressure, heart rate, HbA1c, hemoglobin, creatinine, estimated glomerular filtration rate (eGFR), triglycerides and total cholesterol and its fractions. eGFR was calculated following the Chronic Kidney Disease Epidemiology Collaboration equation and based on the serum creatinine levels [11].

### Participant’s safety

Participants were actively asked about possible adverse events in each visit of the trial and the participant’s medical journal was examined. All adverse effects, regardless of relationship to study drug or protocol were recorded in the adverse event report form.

### Statistics

According to their distribution, continuous data are summarized as mean and standard deviation or as median and percentile 25th–75th. Categorical data are presented as absolute count and percentage. To test differences in changes between baseline and follow-up, new delta variables were computed. To test differences between treatment groups for those delta variables a Student T-test was used whenever data distributed normally and Fisher’s exact test, Mann–Whitney U test or Chi^2^ test was used whenever data was not normally distributed. A two-sided p-value of less than 0.05 was considered statistically significant. All analyses were performed using IBM SPSS Statistics software, version 28.0. Armonk, NY: IBM Corp.

#### Sample size calculation

Initially, we calculated to investigate 84 patients (42 in each arm) to be able to demonstrate a mean absolute difference of 5 percentage units (specifically 90% vs 95%) in strut coverage with an alpha error of 5% (two sided) and a power of 80%. The chosen effect size is 5 percentage units of stent strut coverage as this value has been observed as a clinically relevant threshold to be related with stent-failure outcomes [12]. The sample size calculation was performed estimating a standard deviation of 8% of strut coverage from previous clinical data [13]. We foresaw that some subjects would drop out, and therefore aimed to investigate 100 subjects.

## Results

### Baseline data

At baseline, groups were well balanced regarding clinical characteristics (Table 1). The mean age was 66.6 years (8.8 years) and 58% of the subjects were men. The median duration of diabetes was 3.0 years (1.0–8.0 years) in the exenatide group and 3.5 years (0.6–11.5 years) in the control group. At baseline, mean HbA1c was 60.2 mmol/mol (18.7 mmol/mol) [DCCT 7.7% (1.9%)] in exenatide group and 58.8 mmol/mol (14.0 mmol/mol) [DCCT 7.5% (1.4%)] in the control group. The indication for PCI was an acute coronary syndrome in 45% of the people in the exenatide group and 61% in the control group. The left anterior descendent artery was the most frequently stented vessel both in the exenatide group (58.0%) and in the control group (61.1%). Neither the number of stents implanted per lesion, nor the stent diameter differed between groups.

**Table 1:** Baseline clinical characteristics of the randomized participants

	Exenatide (n = 20)	Control (n = 18)	P value
Age, years	66.0 (58.2–74.7)	63.0 (55.2–68.0)	0.114^a^
Male gender, n (%)	11 (55.0)	11 (61.1)	0.752^b^
Indication for angiography, n (%)			0.483^b^
Stable angina	11 (55.0)	7 (38.9)	
Unstable angina	2 (10.0)	4 (22.2)	
NSTEMI	7 (35.0)	7 (38.9)	
Heredity for CV disease, n (%)	1 (5.0)	5 (27.8)	0.177^b^
Current smoking, n (%)	3 (15.0)	2 (11.1)	0.424^b^
Hypertension, n (%)	14.0 (70.0)	14 (77.8)	0.719^b^
Dyslipidemia, n (%)	8 (42.1)	9 (50.0)	0.746^b^
Diabetes duration, years	3.0 (1.0–8.0)	3.5 (0.6–11.5)	0.973^a^
Heart failure, n (%)	5 (25.0)	3 (16.7)	0.697^b^
Myocardial infarction, n (%)	6 (30.0)	6 (33.3)	1.000^b^
CABG, n (%)	3 (15.0)	3 (5.6)	0.606^b^
Simplex retinopathy, n (%)	2 (10.0)	1 (5.6)	1.000^b^
Proliferative diabetic retinopathy, n (%)	1 (5.0)	1 (5.6)	1.000^b^
Diabetic foot, n (%)	0 (0.0)	1 (5.6)	0.474^b^
Stroke, n (%)	3 (15.0)	1 (5.6)	0.606^b^
Atrial fibrillation, n (%)	4 (20.0)	2 (36.0)	0.107^b^
Betablockers, n (%)	15(75.0)	8 (44.4)	0.096^b^
ACEi/ARB, n (%)	12 (60.0)	15 (83.3)	0.160^b^
Statines, n (%)	16 (80.0)	13 (72.2)	0.709^b^
Anticoagulation, n (%)	4 (20.0)	2 (36.0)	0.107^b^
Metformin, n (%)	19 (95.0)	16 (88.9)	0.595^b^
Insulin, n (%)	8 (40.0)	7 (38.9)	0.564^b^
Long-acting insulin, n (%)	7 (35)	6 (33.3)	1.000^b^
Mixed insulin, n (%)	4 (20)	3 (16.7)	1.000^b^
Sulphonylurea, n (%)	0 (0.0)	1 (5.6)	0.309^b^
Thiazolidinediones, n (%)	0 (0.0)	0 (0.0)	-
SGLT-2-inhibitors, n (%)	0 (0.0)	1 (5.6)	0.309^b^
Weight, kg	93.5 (20.8)	88.1 (12.6)	0.742^c^
BMI, kg/m^2^	32.0 (30.0–33.0)	29.0 (26.4–31.2)	0.164^a^
Abdominal circumference, cm	114.5 (107.0–123.0)	108.0 (102.0–117.7)	0.203^a^
HbA1c, mmol/mol	60.2 (18.7)	58.8 (14.0)	0.783^c^
HbA1c, % (DCCT)	7.7 (1.7)	7.5 (1.3)	0.787^c^
Hemoglobin, g/L	145.0 (123.7–149.5)	140.0 (132.0–149.0)	0.869^a^
Creatinine, µmol/L	77.0 (14.0)	78.4 (16.0)	0.766^c^
eGFR, ml/min/1,73 m	80.5 (14.4)	84.2(14.6)	0.457^c^
Total cholesterol, mmol/L	4.3 (1.5)	3.9 (1.2)	0.410^c^
LDL cholesterol, mmol/L	1.8 (1.2–3.5)	1.6 (1.2–2.5)	0.427^a^
HDL cholesterol, mmol/L	1.1 (0.8–1.2)	1.0 (0.8–1.6)	0.848^a^
Triglycerides, mmol/L	1.7 (1.2–3.0)	1.7 (1.3–2.5)	0.826^a^
Systolic blood pressure, mmHg	138.0 (24.0)	125.0 (18.0)	0.091^c^
Diastolic blood pressure, mmHg	75.0 (13.0)	77.0 (11.0)	0.644^c^
Heart Rate, bpm	68.0 (10.0)	73.0 (14.0)	0.168^c^
Segment in coronary tree, n (%)			0.183^b^
Left Main	3 (15.8)	0 (0.0)	
LAD	11(58.0)	11 (61.1)	
LCX	3 (15.8)	3 (16.7)	
RCA	2 (10.5)	4 (22.2)	
Number of stents, n	1.0 (1.0–2.0)	1.5 (1.0–2.0)	0.331^a^
Stent diameter, mm	3.5 (3.0–4.0)	3.5 (3.3–4.0)	0.687^a^
LVEF by echocardiography, %	55.7 (6.3)	54.7 (11.0)	0.742^c^

Descriptive data is showed as mean (standard deviation) or median (percentile 25th-75th) depending on data distribution. Tested with ^a^Mann- Whitney U, ^b^Fisher’s exact test or Chi2 test or ^c^Student’s T-test. *ACEi/ARB* Angiotensin-converting enzyme inhibitors/angiotensin II receptor blockers, *BMI* Body Mass Index, *CABG* Coronary Artery By-pass Grafting, *CV* Cardiovascular, *DCCT* Diabtes Control and Complications Trial, *eGFR* CKD-EPI Glomerular filtration Rate, *HbA*_*1C*_ glycosylated hemoglobin, *LVEF* left ventricle ejection fraction, *SGLT-2* Sodium-Glucose Co-transporter-2

The OCT findings at baseline were similar between treatment groups (Table 2). The cross-sectional lumen area at maximal obstruction (post-stenting) was 6.1 mm^2^ (1.8 mm^2^) in the exenatide group and 5.1 mm^2^ (1.8 mm^2^) in the control group. The mean stent expansion was 116.6% (24.0%) in the exenatide group and 115.9% (18.7%) in the control group. In addition, the median stent expansion at the smallest stent cross-sectional area was 75.1% (57.4–87.8%) in the exenatide group and 70.4% (57.7–86.9%) in the control group. The presence of calcium in the site of minimum stent area was 57.9% in the exenatide group and 29.4% in the control group (p = 0.106). The presence of significant residual malapposition (i.e. > 300 µm) did not differ between treatment groups (exenatide group 0.79% (1.5%) vs. control group 1.5% (2.7%), (p = 0.347)).

**Table 2:** Optical Coherence Tomography findings at baseline in the reference segments, the stenosis segment and at strut level

	Exenatide (n = 19)	Control (n = 17)	P value
Stent length by OCT, mm	26.3 (20.0–31.4)	28.6 (24.0–40.0)	0.241^a^
Number of frames per pullback, n	539 (374–540)	539 (374–540)	0.742^a^
Reference segments			
CS lumen area proximal reference, mm2	9.6 (7.6–12.6)	8.7 (5.9–11.0)	0.158^a^
Minimal Lumen Diameter at proximal reference, mm	3 (2.7–3.5)	3 (2.5–3.5)	0.384^a^
CS lumen area distal reference, mm2	5.7 (4.6–8.5)	5 (3.3–6.9)	0.334^a^
Minimal Lumen Diameter at distal reference, mm	2.6 (2–3.1)	2.3 (1.8–2.8)	0.261^a^
Dissection at proximal stent edge, n (%)	0 (0.0)	1 (5.9)	0.472^b^
Mean dissection length at proximal stent edge, mm	0 (0.0)	0.05 (0.2)	0.290b
Dissection at distal stent edge, n (%)	2 (10.5)	1 (5.9)	1.000^b^
Mean dissection’s length at distal stent edge, mm	8.4 (7.7–15)	12.1 (7.5–13.2)	0.667^a^
Stenosis segment			
CS lumen area at maximal obstruction, mm2	6.1 (1.8)	5.1 (1.8)	0.122c
Lumen area stenosis (mean area as reference), %	23.2 (3.6–32.7)	17.3 (10–27.8)	0.692^a^
CS stent area at maximal obstruction, mm2	6.1 (1.8)	5.1 (1.8)	0.097^c^
MSA in proximal stent half segment, mm2	7.03 (1.8)	5.9 (1.8)	0.073^c^
MSA in distal stent half segment,	6.1 (2.0)	4.8 (1.7)	0.107^c^
Mean in-stent CS area, mm2	8.1 (2.2)	7 (2.0)	0.134^c^
Stent expansion at MSA, %	75.1 (57.4–87.8)	70.4 (57.7–86.9)	0.704^c^
Mean stent expansion, %	116.6 (24.0)	115.9 (18.7)	0.784^c^
In-stent stent volume, mm3	157.4 (136–273)	166.5 (104.8–258)	0.537^a^
Vessel characterization at MSA			
Plaque characterization			0.280^b^
Fibrotic,n (%)	0 (0.0)	0 (0.0)	
Lipidic, n (%)	4 (21.1)	5 (29.4)	
Mixed non-calcified, n (%)	4 (21.1)	7 (41.2)	
Mixed calcified, n (%)	9 (47.4)	3 (17.6)	
Calcification arc, degrees	65.6 (71)	30.6 (51.5)	0.104^c^
Calcification deep, mm	0.4 (0.4)	0.2 (0.4)	0.340^c^
Thrombus presence, n (%)	7 (36.8)	2 (11.8)	0.128^b^
Tissue prolapse, n (%)	10 (52.6)	8 (47.1)	1.000^b^
Major tissue prolapse, n (%)	3 (15.8%)	3 (17.6%)	0.895^b^
Strut analysis			
Total number of struts analyzed per lesion, n	2031 (664)	2545.5 (1187)	0.113^c^
Number of struts per cross-section, n	10.2 (1.6)	10.3 (1.4)	0.863^c^
Frequency of malapposed struts per lesion, %	5.7 (1.9–12)	5.7 (3.8–13)	0.862^a^
Malapposition > 300 µm,%	0.79 (1.5)	1.5 (2.7)	0.347^c^
Malapposition volume, mm3	11 (4.8)	13.5 (8.4)	0.285^c^
Maximal consecutive length of malapposed struts, mm	1.9 (1.0–3.4)	2 (1.1–3.5)	0.715^a^

OCT findings at baseline directly after stenting. Tested with ^a^Mann-Whitney U test, ^b^Fisher’s exact test or Chi^2^ test, ^c^Student’s T-test. *CS* cross-sectional, *MSA* minimal stent area, *OCT* Optical Coherence Tomography

### Primary outcome

The median percentage of strut coverage did not differ between groups: exenatide group 95.0% (88.7–98.5%) vs. 91.4% (88.8–98.5%) in the control group, (p = 0.692), Table 3 and Fig. 2. To note, there were no statistically significant differences between groups neither in the total number of struts analyzed, nor in the mean number of struts per cross-section (Table 3).

**Table 3:** Changes on Optical Coherence Tomography derived endpoints between baseline and follow-up

	Baseline			Follow-up			Change between baseline and follow up		P value
	Exenatide (n = 19)	Control (n = 17)	P value	Exenatide (n = 16)	Control (n = 17)	P value	Exenatide (n = 15)	Control (n = 17)	P value
Strut analysis									
Covered struts per lesion, %	–	–	–	95.0 (88.7–98.5)	91.4 (88.8–98.5)	0.692^a^	–	–	–
Covered struts > 40 µm, %				79.4 (35.0–95.0)	64 (34.3–90.5)	0.589^a^			
Malapposition > 300 µm,%	0.8 (1.5)	1.5 (2.7)	0.347^b^	0.4 (0.8)	1.4 (4.2)	0.368^b^	− 0.2 (1.2)	− 0.0 (2.5)	0.829^b^
Malapposition volume, mm^3^	11 (4.8)	13.5 (8.4)	0.285^b^	2.2 (2.6)	3.7 (5.3)	0.322^b^	− 9 (5.4)	− 9.2 (7.5)	0.892^b^
Mean NIH area, mm^2^	–	–	–	1.3 (0.4)	1.1 (0.5)	0.423^b^	–	–	–
NIH area at maximal obstruction, mm^2^	–	–	–	1.2 (1–2.3)	1.1 (0.7–1.9)	0.407^a^	–	–	–
NIH stenosis, %	–	–	–	15.5 (10.6–19.2)	14.7 (11–17.1)	0.801^a^	–	–	–
NIH volume at maximal obstruction, mm3	–	–	–	33.8 (20)	33.8 (17.4)	1.000^b^	–	–	–
Maximal neo-intimal thickness at maximal obstruction, mm	–	–	–	0.2 (0.1–0.3)	0.2 (0.1–0.6)	0.471^a^	–	–	–
Total number of struts analyzed per lesion, n	2031 (664)	2545.5 (1187)	0.113^b^	2154 (924)	2579 (1380)	0.304^b^			
Number of struts per cross-section, n	10.2 (1.6)	10.3 (1.4)	0.863^b^	10.9 (1.6)	10.6 (1.6)	0.690^b^			
Stenosis segment									
Minimum lumen diameter at maximal obstruction, mm	2.4 (0.4)	2.3 (0.5)	0.464^b^	2.3 (0.5)	2.1 (0.6)	0.517^b^	− 0.2 (0.4)	− 0.2 (0.4)	0.798^b^
Mean in-stent minimal lumen diameter, mm	2.9 (0.4)	2.7 (0.4)	0.261^b^	2.8 (0.4)	2.7 (0.5)	0.382^b^	− 0.1 (0.2)	− 0.0 (0.2)	0.165^b^
CS lumen area at maximal obstruction, mm^2^	6.1 (1.8)	5.1 (1.8)	0.122^b^	5 (2)	4.4 (2.2)	0.362^b^	− 0.6 (− 0.2–1.6)	− 0.6 (0.3–1.0)	0.635^a^
Lumen area stenosis (mean area as reference), %	23.2 (3.6–32.7)	17.3 (10–27.8)	0.692^a^	30.4 (17.7–46.3)	32.7 (18.2–46.1)	0.857^a^	13.7 (3.6–24.6)	12.6 (− 5–29.5)	0.813^a^
CS stent area at maximal obstruction, mm^2^	6.1 (1.8)	5.1 (1.8)	0.097^b^	6.6 (2.1)	5.8 (2.1)	0.272^b^	0.2 (1.8)	0.9 (1.0)	0.231^b^
Mean in-stent CS area, mm^2^	8.1 (2.2)	7 (2.0)	0.134^b^	7.6 (1.9)	6.9 (2.7)	0.885^b^	0.3 (1)	1.1 (1.0)	0.054^b^
In-stent lumen volume, mm^3^	204.7 (155.8–296)	192.2 (154.9–283.7)	0.937^a^	246.8 (155.3–276)	187.1 (123.6–289)	0.756^a^	− 17.8 (− 62.2–20.1)	− 2.6 (− 34–62.4)	0.268^a^
In-stent stent volume, mm^3^	157.4 (136–273)	166.5 (104.8–258)	0.537^a^	228.9 (136.1–297.3)	206.9 (145.3–312.3)	0.971^a^	13.4 (-36.3–71)	48.5 (27.3–140.5)	0.048^a^

Differences between groups at baseline, follow-up and in changes between baseline and follow-up in OCT-derived endpoints. Tested with ^a^Mann-Whitney U test, ^b^Student’s T-test. *CS* cross-sectional, *NIH* neo-intima hyperplasia

### Secondary outcomes

#### OCT derived secondary outcomes

Strut coverage with neo-intimal thickness > 40 µm did not differ between groups: exenatide group 79.4% (35–95%) vs. 64% (34.3–90.5%) in the control group (p = 0.589). The percentage of relevant malapposition (i.e. > 300 µm) was low and not significantly different between treatment groups: exenatide group 0.4% (0.8%) and 1.4% (4.2%) in the control group (p = 0.368). The mean area of neo-intimal hyperplasia was 1.3 mm^2^ (0.4 mm^2^) in the exenatide group and 1.1 mm^2^ (0.5 mm^2^) in the control group (p = 0.423). The neo-intimal hyperplasia stenosis was 15.5% (10.6–19.2%) in the exenatide group and 14.7% (11–17.1%) in the control group. These and the rest of OCT derived secondary endpoints are summarized in Table 3 and Fig. 2. Vessel wall characterization at baseline for the reference segments is found in Additional file 1: Table S4.

#### Clinical secondary outcomes

There was a significantly greater reduction in body weight and HbA1c between groups: exenatide group − 7.8 kg (19.7 kg) compared to control group 0.4 kg (4.1 kg), (p = 0.014), and − 11.4 mmol/mol (10 mmol/mol) [DCCT − 1.1% (1%)] compared to control group -4.7 mmol/mol (11.1 mmol/mol) [DCCT − 0.4% (1.1%)], (p = 0.001), respectively. No relevant differences between groups were found for blood lipid levels, renal function, or the rest of the clinical secondary endpoints (Table 4).

**Table 4:** Changes between baseline and follow up for the clinical endpoints

	Exenatide (n = 16)	Control (n = 17)	P value
Weight, Kg	− 7.8 (19.7)	0.4 (4.1)	0.014^a^
Abdominal circumference, cm	− 3 (− 9.0 to − 1.0)	− 0.9 (− 6.0 to 5.0)	0.249^b^
HbA1c, mmol/mol	− 11.4 (10.0)	− 4.7 (11.1)	0.001^a^
Hemoglobin, g/L	− 3.0 (− 9.0 to 3.0)	1.0 (− 8.2 to 5.5)	0.363^b^
Creatinine, mg/dl	0.04 (0.15)	0.03 (0.17)	0.946^a^
eGFR, ml/min/1,73m2	− 3.0 (13.1)	− 1.8 (10.3)	0.772^a^
Cholesterol, mmol/L	− 1.04 (0.9)	− 0.3 (0.9)	0.144^a^
LDL cholesterol, mmol/L	− 0.7 (− 1.6 to 0.2)	− 0.5 (− 0.8 to 0.1)	0.261^b^
HDL cholesterol, mmol/L	− 0.5 (− 0.3 to 0.1)	0.1 (0.1 to 0.2)	0.117^b^
Triglycerides, mmol/L	− 0.3 (− 1.1 to 0.1)	− 0.1 (− 0.7 to 0.7)	0.140^b^
Systolic blood pressure, mmHg	− 4.5 (24.6)	2.0 (17.6)	0.441^a^
Diastolic blood pressure, mmHg	− 0.8 (18.7)	− 2.3 (12.1)	0.805^a^
Heart rate, beats per minute	8.0 (7.7)	− 3.3 (12.5)	0.008^a^
Target lesion revascularization, n (%)	0 (0.0)	3 (20.0)	0.224^c^

Differences between groups for changes between baseline and follow-up for the clinical endpoints. Tested with

^a^Student’s T test, ^b^Mann-Whitney U, ^c^Fisher’s exact test. eGFR, CKD-EPI glomerular filtration rate; *HbA1c* glycosylated hemoglobin

#### Adverse events and drop-outs

There were no serious adverse events during the study neither from the study drug or the follow-up invasive procedure. Adverse events and drop-outs are summarized in Additional file 1: Table S5.

## Discussion

### Stent coverage and neo-intima hyperplasia

In the present study we found no evidence that the GLP-1R agonist exenatide improves coronary stent endothelization in subjects with T2D. Since the same type of stent was implanted in all subjects and that the baseline clinical data and the OCT procedural characteristics were well balanced, we consider the comparison between groups reliable. Despite no significant difference in metformin and insulin doses during the study, glycemic control and weight loss were improved in the exenatide group compared to control group. Although the role of glycemic control in subjects with diabetes and DES endothelization remains elusive, [14] activation of GLP-1R may evoke endothelization beyond glycemic control [5–7].

Procedural baseline characteristics, e.g. good stent expansion and the observed low frequency of significant malapposition by OCT, may have prompted an optimal stent endothelization in both groups as the percentage of covered struts is somehow higher than in previous rapports [15]. In the present study strut coverage was analyzed as any coverage, and coverage with a neo-intimal thickness of at least 40 µm. It has been suggested that the presence of underlying vascular smooth muscle cells and a proteoglycan–collagen rich matrix is required to regain a competent endothelium barrier [16]. A recent ex vivo human autopsy OCT study revealed that a neo-intimal thickness of 40 µm, or more, is the most accurate cutoff value to identify complete strut coverage as assessed by histology with a sensitivity of 99.3% and a specificity of 91% [16]. Interestingly, the median strut coverage with neo-intima thickness more than 40 µm was higher in the exenatide group compared to the control group. However, this difference was not statistically significant, and it is unclear whether this could be a signal of a possible effect. Furthermore, it is not known whether if a histologically complete endothelial layer translates into a functionally competent barrier conferring lower risk for stent failure. However, being this a potentially clinically meaningful finding further focused studies are needed.

GLP-1R activation by exenatide has been demonstrated inducing endothelial proliferation, [5] and reduces intima hyperplasia [7]. Neo-intima hyperplasia was detected by OCT, and, despite luminal measurements decreased slightly at follow-up (due to neo-intima proliferation) in both groups, however without any obstruction; there were no significant differences in luminal measurements, nor in neo-intima thickness, between groups.

### Clinical findings

There was no difference in the need for target lesion revascularization, nor in the amount of intima hyperplasia detected by OCT, between groups. In contrast, subjects treated with exenatide had a greater weight loss and decreased abdominal circumference, and a corresponding better glycemic control, compared to the control group; supporting the role on weight reduction and glycemic control for incretin treatment in people with T2D. Drop-outs were more frequent in the exenatide group (4 participants) than in the control group (1 participant), which were related to the study drug in two cases, i.e. one subject suffered from diarrhea and nausea and the other subject of pain during the subcutaneous injection administration. To note, one of the patients that discontinued the treatment with exenatide did complete the follow-up period and was analyzed for strut coverage as an intention to treat analysis.

### Significance

Opposed to native vessel atherosclerosis, neo-atherosclerosis is a process that may rapidly develops (within months) characterized by the presence of lipid-laden macrophages, within the neo-intima layer, secondary to dysfunctional endothelial coverage of the stent. Following a PCI, it is therefore of major interest to achieve a quick and functionally mature neo-intima layer covering the entire stent. This should be kept in mind since ISR not only is a problem because of angina relapse, but also since PCI in a restenosed vessel is associated with even a higher risk of major adverse cardiovascular events (MACE) than de novo PCI [17]. Additionally, ISR and ST are independent predictors of MACE and frequently present as an acute coronary syndrome [18]. Despite beneficial effects, observed in preclinical studies, of the activation of GLP-1R on the endothelium [5–7] and with Dipeptidyl Peptidase- 4 inhibitor eluting stents [19] the present trial could not confirm any evidence of stent coverage, or reduced neo-intima thickness. Also, our findings are in line with a previous register-based study where no reductions on the risk of ISR or ST were found in T2D subjects with modern DES while treated with incretin-based therapy [20]. In contrast, other antidiabetic treatment has shown beneficial action on this issue since it recently was demonstrated that Sodium-Glucose Co-transporter-2 (SGLT-2) inhibitors reduced the risk of ISR-driven MACE in subjects with T2D in an observational study; [21] a finding that has to be confirmed in a randomized clinical trial.

## Strengths and limitations

To the best of our knowledge the present study is the first randomized trial to specifically address the effect of a GLP-1R agonist on human coronary stent endothelization using OCT. Moreover, no previous human study has reported on the effect of GLP-1RA on the occurrence of neo-atherosclerosis in subjects with T2D. However, there are several limitations. Firstly, we did not reach the number of participants guided from the sample size calculation and therefore the study may have been underpowered. The sample size calculation of the present study was based on in the OCTAMI trial; [13] although not entirely reflecting the population in the present study, it was the most appropriate at the time of trial planning and protocol writing. During the following years several recent studies [15, 22–24] have included participants that better represent the current study population, from which hypothetical sample size recalculations would have ranged from 18 to 98 individuals. The reasons for the lower inclusion are shared between a higher rate of screening failure than anticipated and the halt in participant inclusion due to COVID-19 pandemic. The main reason for screening failure was that the eligible subject was already treated with an incretin-based therapy as the rate of prescription of these drugs has exponentially grown in Sweden since its introduction in 2006 [20]. The frequency of the different causes for screening failure are summarized in Additional file 1: Table S2. Secondly, strut coverage is a non-clinical endpoint that works as a proxy for ST and ISR. To date, there is no clear prospective evidence of a stent coverage cut off point that would prevent the risk of stent failure. However, the presence of uncovered struts is a frequent finding when imaging is used in cases of late ST [25] and the reconstitution of a complete endothelial barrier is essential to avoid neo-atherosclerosis with subsequent ISR [26]. While neo-intima hyperplasia has historically been described as the main cause of ISR in the BMS era, this histopathological feature is inhibited by the antiproliferative drug that the DES elutes [26]. The inherent consequence of this action is a delayed reconstitution of the endothelial barrier that, in its turn, would trigger the process of neo-atherosclerosis resulting in ISR and even ST when neo-intimal plaque rupture occurs [27]. Given the relatively low frequency of these events and the chronological unpredictability, a clinical endpoint would not have been pragmatic. Thirdly, the timepoint for follow-up can be discussed not at least for the neo-intima hyperplasia and neo-atherosclerosis endpoints. Classically, in the bare metal stent (BMS) era, ISR tended to peak at 6 months post stent-implantation [2]. However, today it is suggested that BMS-ISR and DES-ISR are distinct pathological entities as they may differ in chronology, histopathology and even in the response to intervention. In contrast to BMS-ISR, the risk of DES-ISR seems to continuously increase even years after stent implantation [2]. The yearly rate of DES-ISR has been estimated to 2%.[26] Altogether, 3 months may be a too short period to detect signs of ongoing neo-intima hyperplasia or neo-atherosclerosis. At the time of the design of this study, the difference between BMS and DES regarding the chronology of stent healing and the occurrence neoatherosclerosis was not well described yet. On regard of the timing for the primary endpoint, although strut coverage is known to be delayed with DES compared to BMS because of the targeted effect of the antiproliferative drug [26], previous reports on zotarolimus-DES strut coverage showed high percentages of DES coverage at 12 weeks and even at shorter follow-up time [9, 23]. These findings did encourage the chosen timepoint for follow-up in the present study, but it is legitim to wonder if 12 weeks is brief time for a drug to influence the outcome giving the neutral results of this trial. Furthermore, a clinical assessment of the endothelial function would have been of interest to complement the results on neo-endothelial stent coverage. Finally, we cannot exclude that other GLP-1R analogs would have had different effects on our outcomes of interest. The efficacy on CV outcomes has not been demonstrated for all GLP-1RA. Indeed, the EXSCEL trial [28], where 14752 individuals were randomized to either exenatide or placebo found neutral results for the primary endpoint of MACE (I.e. CV death, non-fatal MI, non-fatal stroke). The GLP-1RA with confirmed CV efficacy are liraglutide [29], semaglutide (subcutaneous administration) [30], albiglutide [31], dulaglutide [32] and efpeglenatide [33]. It is unclear whether the the differences in the observed CV effects between this family of drugs can be truly related to specific actions, trial design or the extent of participant compliance for each trial [34]. In any case, exenatide was chosen for this study due to the promising preclinical data and before the publication of the results of EXSEL trial.

## Conclusion

In this assessor blinded, randomized trial we found no evidence of a favorable effect of exenatide neither on strut coverage of a modern DES, nor on the occurrence of neo-intima hyperplasia, or neo-atherosclerosis in subjects with T2D and coronary artery disease.

### Supplementary Information

**Additional file 1: Table S1** List of exclusion criteria. **Table S2** Reasons for screening failure. **Table S3** OCT endpoints’ definitions. **Table S4** Plaque characterization at reference segments. **Table S5** Adverse events and drop-offs. **Table S6** Glucose lowering medications at follow-up.

## Data Availability

The datasets analyzed during the current study are available from the corresponding author on reasonable request.

## Abbreviations

BMS: Bare metal stent
CERC: European Cardiovascular Research Center
CHD: Coronary heart disease
CV: Cardiovascular
DES: Drug-eluting stent
GLP-1R: Glucagon-like peptide-1 receptor
HbA1c: Glycated hemoglobin
IHD: Ischemic heart disease
ISR: In-stent restenosis
MACE: Major adverse cardiovascular events
NSTEMI: Non-ST elevation myocardial infarction
PCI: Percutaneous coronary intervention
T2D: Type 2 diabetes
SGLT-2: Sodium-Glucose Co-transporter-2
ST: Stent thrombosis
OCT: Optical coherence tomography
