# Supplementary material for: Effects of exenatide on coronary stent’s endothelialization in subjects with type 2 diabetes: a randomized controlled trial. The Rebuild study
Source: Cardiovasc Diabetol. 2023 Dec 8;22:337. doi: 10.1186/s12933-023-02071-4 (PMC10709975; doi:10.1186/s12933-023-02071-4)
Supplement: Supplementary file 1 — Additional file 1: Table S1 List of exclusion criteria. Table S2 Reasons for screening failure. Table S3 OCT endpoints’ definitions. Table S4 Plaque characterization at reference segments. Table S5 Adverse events and drop-offs. Table S6 Glucose lowering medications at follow-up. [file 12933_2023_2071_MOESM1_ESM.docx]

**Title:** Effects of exenatide on coronary stent’s endothelialization in subjects with type 2 diabetes: a randomized controlled trial. The Rebuild study.

Irene Santos-Pardo^a^, MD. Nils Witt^a^, MD, PhD. Oskar Angerås^b^, MD, PhD. Thomas Nyström^c^, MD, PhD.

^a^Department of Clinical Science and Education, Karolinska Institute, Unit of Cardiology, Södersjukhuset, Stockholm, Sweden.

^b^Department of Molecular and Clinical medicine, Institute of Medicine, University of Gothenburg, Gothenburg, Sweden. Department of Cardiology, Sahlgrenska University Hospital, Gothenburg, Sweden

^c^Department of Clinical Science and Education, Karolinska Institute, Unit of Internal Medicine, Södersjukhuset, Stockholm, Sweden

**Additional Material**

Page

Table S1 List of exclusion criteria 2

Table S2 Reasons for screening failure 3

Table S3 OCT endpoints’ definitions 4

Table S4 Plaque characterization at reference segments 6

Table S5 Adverse events and drop-offs 7

Table S6 Glucose lowering medications at follow-up 8

**Table S1 List of exclusion criteria**

| Previous diagnosis of type 1 diabetes |
| --- |
| Current treatment with GLP-1R agonists or DPP-4i |
| Glomerular filtration rate ^a^ <45 ml/min/1.73m^2^ |
| STEMI |
| Significant heart disease besides IHD (significant valvopathy, severe heart failure classified as NYHA 4, active myocarditis, ventricular tachycardia the previous 3 months and untreated atrio-ventricular block type II or III) |
| Malignant neoplasm the previous 5 years requiring surgery, chemotherapy, radiation or palliative therapy |
| History of acute or chronic pancreatitis |
| Current alcohol abuse |
| Pregnancy |

Main exclusion criteria applied to preliminary eligible subjects to participate in the study. ^a^ Calculated with CKD-EPI Glomerular filtration Rate. Abbreviations: DDP4-i, Dipeptidyl Peptidase-4 inhibitors; IHD, Ischemic heart disease; NYHA, New York Heart Association Classification; STEMI, ST-elevation myocardial infarction

**Table S2 Reasons for screening failure**

| **Reason for screening failure** | **n (%)** |
| --- | --- |
| Already treated with incretins | 31(22.8%) |
| eGFR<45ml/min/1.73m^2^ | 17 (12%) |
| Rejects participation | 16 (11.2%) |
| Non-Swedish speaker | 13 (9.1%) |
| Non-significant coronary stenosis | 12 (8.4%) |
| HbA1c<47 mmol/mol | 11 (7.9%) |
| Referred for coronary artery by-pass grafting | 10 (7.0%) |
| Severe heart failure (NYHA 4) | 6 (4.2%) |
| Chronic total occlusion PCI | 6 (4.2%) |
| Ongoing cancer treatment | 2 (1.4%) |
| PCI on ISR | 2 (1.4%) |
| Previous pancreatitis | 2 (1.4%) |
| Other reasons (current alcohol abuse, no reason given) | 14 (10%) |

Reasons for screening failure, absolute numbers and relative frequencies. Abbreviations: eGFR, CKD-EPI Glomerular filtration Rate; HbA1C, glycosylated hemoglobin; NYHA, New York Heart association classification for heart failure severity, ISR, in-stent restenosis.

**Table S3 OCT endpoints’ definitions**

| **Variable** | **Definition** |
| --- | --- |
| Stent length by OCT, mm | Total number of frames between proximal and distal stent reference point * slice thickness (mm) |
| Minimal Lumen Diameter at proximal/distal reference or maximal obstruction, mm | The shortest diameter through the center of mass of the lumen at the proximal/distal reference or maximal obstruction point (user defined frame) |
| Lumen eccentricity at proximal/distal reference or maximal obstruction | Maximum lumen diameter - minimum lumen diameter/maximum lumen diameter at the proximal/distal reference or maximal obstruction point |
| CS lumen area proximal/distal reference or maximal obstruction, mm^2^ | The area bounded by the luminal border at the proximal/distal reference or maximal obstruction point |
| Lumen area stenosis, % | Reference lumen area - minimum lumen area/reference lumen area at the reference point |
| CS stent area at maximal obstruction, mm^2^ | The area bounded by the stent border at the maximal obstruction point |
| Mean in-stent CS area, mm^2^ | Average of CS lumen areas calculated for every frame within start and end frame for the stent |
| MSA in proximal stent half segment, mm^2^ | The smallest stent area within the half proximal segment of the stent. |
| MSA in distal stent half segment, | The smallest stent area within the half distal segment of the stent. |
| Stent expansion at MSA, % | MSA / ((proximal reference area + distal reference area)/2)*100 |
| Mean stent expansion, % | Mean stent CS area / ((proximal reference area + distal reference area)/2)*100 |
| In-stent lumen volume, mm^3^ | Sum of all lumen areas within stent region*slice thickness (mm) |
| In-stent stent volume, mm^3^ | Sum of all stent areas*slice thickness (mm) |
| **Strut analysis** | |
| Total number of struts analyzed per lesion, n | Sum of all analyzed struts |
| Number of struts per cross-section, n | Sum of all analyzed struts / number of frames within stent |
| Covered struts per lesion, % | (Number of covered struts / total number of struts)*100 |
| Covered struts >40 µm, % | (Number of covered struts with >40 µm of tissue in the abluminal part of the strut / total number of struts)*100 |
| Malapposition | Distance from luminal side of strut edge to lumen border greater than strut thickness (81 µm) |
| Malapposition >300µm | Distance from luminal side of strut edge to lumen border greater than 300 µm |
| Frequency of malapposed struts per lesion, % | (Number of malapposed struts / total number of struts)*100 |
| Malapposition >300µm,% | (Number of malapposed struts more than 300 µm / total number of struts)*100 |
| Malapposition volume, mm^3^ | Sum of all stent areas with malapposed struts *slice thickness (mm) |
| Maximal consecutive length of malapposed struts, mm | Total number of consecutive frames with at least one strut identified as malapposed*slice thickness (mm) |
| Mean NIH area, mm^2^ | Sum of NIH areas / number of frames with NIH areas |
| NIH area at maximal obstruction, mm^2^ | NIH area at the point of maximal obstruction |
| NIH stenosis, % | Mean stent CS area - luminal CS area at maximal obstruction by NIH / mean stent CS area. |
| NIH volume at maximal obstruction, mm3 | Amount of plaque within stent lumen area at maximal obstruction * slice thickness (mm) |
| Maximal NIH thickness at maximal obstruction, mm | Maximal distance between NI and stent area measured (manually) at the point of maximal obstruction |

Definitions for all the reported OCT endpoints. Abbreviations: CS, cross-sectional; MSA, minimal stent area; NI, neo-intima; NIH, neo-intima hyperplasia; OCT, optical coherence tomography.

**Table S4 Plaque characterization at reference segments**

|  | **Exenatide (n=19)** | **Control (n=17)** | **P value** |
| --- | --- | --- | --- |
| Lumen eccentricity in proximal reference, mm | 0.2 (0.1-0.3) | 0.1 (0.1-0.2) | 0.051^a^ |
| Plaque characterization at proximal reference | | | 0.840 ^b^ |
| Fibrotic,n (%) | 0 (0) | 0 (0) |  |
| Lipidic, n (%) | 5 (35.7) | 3 (25) |  |
| Mixed non-calcified, n (%) | 5 (35.7) | 5 (41.7) |  |
| Mixed calcified, n (%) | 4 (28.6) | 4 (33.3) |  |
| **Distal reference segment** | | | |
| Lumen eccentricity in distal reference, mm | 0.1 (0.1-0.2) | 0.1 (0.1-0.2) | 0.235^b^ |
| Plaque characterization at proximal reference | | | 0.337 ^b^ |
| Fibrotic,n (%) | 1 (5.9) | 4 (26.7) |  |
| Lipidic, n (%) | 5 (29.4) | 5 (33.3) |  |
| Mixed non-calcified, n (%) | 8 (47.1) | 5 (33.3) |  |
| Mixed calcified, n (%) | 3 (17.6) | 1 (6.7) |  |

Wall vessel characterization at reference segments (within 5 mm from the proximal and distal stent edge). ^a^Tested with Mann-Whitney U test, ^b^tested with Chi ^2^ test.

**Table S5 Adverse events and drop-offs**

| **Treatment group** | **Adverse effect** | **Medical consequence** | **Trial participation discontinuation** |
| --- | --- | --- | --- |
| Exenatide | Lumps on injection site | None | No |
| Exenatide | Lumps on injection site | None | No |
| Exenatide | Nausea and Diarrhea | Stopped exenatide injections | Yes |
| Exenatide | Chest pain | Hospitalization. No evidence of ongoing myocardial ischemia found. No changes in treatment | No |
| Exenatide | Fail to handle fear for injections | Denied continuing drug administration despite education on injection was offered | Yes |
| Exenatide | No special reason given | - | Yes |
| Exenatide | No special reason given | - | Yes |
| Control | Cutaneous rash | None | No |
| Control | Chest pain | Hospitalization. NSTEMI. Referred for coronary artery by-pass grafting | Yes |

Adverse effects and drop-offs for each treatment group.

**Table S6 Glucose lowering medications at follow-up**

| **Drug class** | **Exenatide (n=17)** | **Control (n=17)** | **P value** |
| --- | --- | --- | --- |
| Insulin at follow up, n (%) | 13 (76.5) | 13 (76.5) | 1.000^a^ |
| Total units of mixed insulin per day | 5.2 (8.9) | 2.6 (4.2) | 0.299^b^ |
| Total units of long-acting insulin per day | 8.1 (11.2) | 1.8 (0.8) | 0.185^b^ |
| Metformin at follow up, n (%) | 17 (100) | 14 (82.4) | 0.227^a^ |
| Total grams of metformin per day | 1.0 (1.0-2.0) | 2.0 (1.5-2.0) | 1.000^c^ |
| SGLT-2-inhibitors, n (%) | 0 (0) | 1 (5.9) | 1.000^a^ |
| Sulphonylurea, n (%) | 0 (0) | 1 (5.9) | 1.000 ^a^ |
| Thiazolidinediones, n (%) | 0 (0) | 0 (0) | - |

Glucose lowering medications at follow-up for each treatment group. Tested with ^a^Fisher’s exact test, ^b^Student’s T test,^c^Mann-Whitney U test. Abbreviations: SGLT-2 inhibitors, sodium-glucose Co-transporter-2 inhibitors.
